# On the effects of signal processing on sample entropy for postural control

**DOI:** 10.1371/journal.pone.0193460

**Published:** 2018-03-01

**Authors:** Anat V. Lubetzky, Daphna Harel, Eyal Lubetzky

**Affiliations:** 1 Department of Physical Therapy, Steinhardt School of Culture Education and Human Development, New York University, New York, NY, United States of America; 2 Department of Applied Statistics, Social Science and Humanities, Steinhardt School of Culture Education and Human Development, New York University, New York, NY, United States of America; 3 Courant Institute of Mathematical Sciences, New York University, New York, NY, United States of America; University of Illinois at Urbana-Champaign, UNITED STATES

## Abstract

Sample entropy, a measure of time series regularity, has become increasingly popular in postural control research. We are developing a virtual reality assessment of sensory integration for postural control in people with vestibular dysfunction and wished to apply sample entropy as an outcome measure. However, despite the common use of sample entropy to quantify postural sway, we found lack of consistency in the literature regarding center-of-pressure signal manipulations prior to the computation of sample entropy. We therefore wished to investigate the effect of parameters choice and signal processing on participants’ sample entropy outcome. For that purpose, we compared center-of-pressure sample entropy data between patients with vestibular dysfunction and age-matched controls. Within our assessment, participants observed virtual reality scenes, while standing on floor or a compliant surface. We then analyzed the effect of: modification of the radius of similarity (*r*) and the embedding dimension (*m*); down-sampling or filtering and differencing or detrending. When analyzing the raw center-of-pressure data, we found a significant main effect of surface in medio-lateral and anterior-posterior directions across *r*’s and *m*’s. We also found a significant interaction group × surface in the medio-lateral direction when *r* was 0.05 or 0.1 with a monotonic increase in *p* value with increasing *r* in both *m*’s. These effects were maintained with down-sampling by 2, 3, and 4 and with detrending but not with filtering and differencing. Based on these findings, we suggest that for sample entropy to be compared across postural control studies, there needs to be increased consistency, particularly of signal handling prior to the calculation of sample entropy. Procedures such as filtering, differencing or detrending affect sample entropy values and could artificially alter the time series pattern. Therefore, if such procedures are performed they should be well justified.

## Introduction

Approximate entropy (ApEn) was developed by Pincus to quantify the amount of regularity of fluctuations in time-series data. Regularity of physiological signals (e.g., heart rate tracing) was suggested as an indication of biological systems’ health [1]. Sickness and aging were found to be associated with decreased ApEn values indicating more regularity [2]. As a refinement of this algorithm, Richman and Moorman [3] introduced Sample Entropy (SampEn), defined as the negative natural logarithm of the probability that two uniformly chosen distinct vectors of *m* + 1 consecutive data points have distance *r*, given that their *m*-long prefixes have distance at most *r* (the parameter *m* is referred to as the embedding dimension, and *r* is the tolerance window, or radius of similarity). Richman and Moorman suggested that SampEn has several advantages over ApEn including faster computing time, and better consistency across *m* and *r* when the number of data points, *N*, is sufficiently large [3].

Since its introduction in 2000, SampEn has been used to study postural control given various experimental conditions [4,5], to assess effects of disease or age [6,7], as an indication of balance [8] or to compare performance before and after interventions [4,9]. Nevertheless, large variability in the handling of the signal and the choice of parameters makes the comparison between studies unfeasible.

Addressing the choice of parameters (*r*, *m*), Yentes et al. found *m* = 2 to be optimal for gait and theoretical data [10]. Postural control studies typically utilized either *m* = 2 (e.g., [5,9,11]) or *m* = 3 (e.g., [4,6,8]). Yentes et al. also suggested that, for gait data, the radius of similarity should be examined per the experimental conditions. While *r*’s between 0.1 and 0.25 (multiplied by the standard deviation of the signal) have commonly been used in the literature [5,10], *r* = 0.3 have also been reported [8]. Richman and Moorman [3] observed that, on a data sequence of independent uniform random variables on [0,1], the mean of SampEn decreased roughly as log(1/*r*) when *r* increased. The behavior of the *average* value of SampEn in a population across different values of *m* and *r* has been studied before, particularly in gait data. A fundamental question that we wished to address is the following: if one can statistically distinguish between the *distributions* of SampEn in 2 different populations for one common set of parameters, would that also be the case for other popular choices of *m* and *r*. To that purpose, exploration of the variance in a population is warranted beyond the SampEn mean.

Another important question for the application of SampEn to postural control research is whether the time series should be filtered prior to the application of SampEn such as done in ‘traditional’ analysis of sway magnitude and velocity. Traditionally, postural control analysis relied on postural stability (i.e., the ability to resist perturbations) and postural steadiness (i.e., the ability to stand as motionless as possible) as indications of balance control [12]. In this ‘linear modeling’, variability of the COP location during quiet standing was considered to be random error. Because human postural sway has been shown to be mostly under 2 Hz [13], studies utilizing linear parameters such as directional path or root mean square of velocity typically sample at 100 Hz [14–16], but higher sampling frequencies of COP have also been reported (e.g., 1000 Hz [17] or even 2400 Hz [18]). A low-pass Butterworth filter at 5 Hz or 10 Hz was then considered a reasonable approach to eliminate noise and frequencies that are outside of human postural performance [16]. When it comes to SampEn, however, it has been suggested that a traditional filter may smooth out the unique features of the time series [19,20]. The literature is diverse here as well with several studies applying a filter [4–6], others applying down-sampling techniques [7,11,21] and others analyzing the original data set [8,9]. These differences in data processing techniques have been shown to impact the value of SampEn, especially following down-sampling to 25 Hz or when SampEn was derived from differenced data [19]. However, their effect on the sensitivity of SampEn to detect differences between group or environmental conditions has not yet been investigated.

Finally, biological data often present non-stationarity (e.g., a drift). Stationarity has not been set as a requirement for SampEn originally [3], and the exact nature of stationarity required for SampEn, as well as other non-linear measures, is unclear [20]. Nevertheless, stationarity was typically being addressed in studies utilizing SampEn for postural control analysis. Several authors suggested that the time-series signal should be differenced prior to the calculation of SampEn [4,6,9,22], i.e., they used the velocity data rather than the original position data. If ${\left(Y_{t}\right)}_{t=0}^{N}$ is a time series, we refer to ${\left(Y_{t}-Y_{t-1}\right)}_{t=1}^{N}$ as its difference series. D’Amico and Ferrigno noted that differencing acts as a high-pass filter and thus amplifies high-frequency noise [23]. Others applied detrending of the signal, i.e., subtracting the best linear approximation (the line achieving the least sum of squares from the original time series) prior to performing calculations [5,7] in addition to filtering [5] or down-sampling [7] the signal. The risk of detrending, however, is the possibility of removing part of the dynamics of the signal [20]. With that, several studies have chosen to calculate SampEn for the original COP position time-series [8,11,21]. Better understanding of what each of these procedures does to the signal and how it affects the SampEn outcome may lead to improved consistency across postural control research.

Given this large procedural variability between studies regarding SampEn and postural control, the purpose of this work was to investigate the effect of parameter selection (*m*’s and *r*’s); signal manipulation (down-sampling and filter) and data pre-processing (detrending and differencing) on sample entropy as an outcome measure to compare clinical groups and environments. We utilized clinical experimental data to investigate the aforementioned questions. Specifically, we tested whether a group of patients with vestibular dysfunction has different regularity than healthy controls given certain visual tasks (visual input provided by the Oculus Rift or a simple ‘eyes closed’ task) and certain somatosensory conditions manipulated by two support surfaces (stable vs. compliant surface). We then tested the effect of the following signal manipulations on the answer to this question: (1) six different levels of *r*’s and 2 levels of *m*’s; (2) four different down-sampling techniques and a traditional Butterworth filter; and (3) pre-processing of the COP signal with differencing or detrending. It was expected that SampEn should be monotone decreasing in *r*, but we hypothesized that the ability to detect differences between groups may be reduced. We expected down-sampling to mildly increase SampEn values because the increased distance between data points may reduce the ability to predict the location of the following data point, however, we expected this effect to be symmetric between the conditions. We also hypothesized that filtering will alter the original patterns. Finally, since the original position is important for identifying the sway pattern and the person is performing a static task, differencing might mask the phenomena observed with the original time series, while detrending, a more conservative approach, may not impact it to the same extent.

## Materials and methods

### Experimental procedures

Adult men and women (18 or older), able to sign an informed consent in English were included. Exclusion criteria were: peripheral neuropathy, uncorrected visual impairment, and pregnancy. Seventeen patients with vestibular dysfunction (mean age = 57.82, SD = 18.18, minimum 24, maximum 81, 10 women,) and 11 healthy controls (mean age = 52.9, SD = 18.03, minimum 24, maximum 79, 6 women) were tested. Eligible patients were clinically diagnosed with peripheral bilateral or unilateral, acute or chronic vestibular dysfunction. Patients were recruited from the Vestibular Rehabilitation Clinic of the New York Eye and Ear (NYEE) Infirmary of Mount Sinai and controls were recruited from the general community. This study was approved by the Institutional Review Boards of the NYEE and New York University.

The rationale and motivation for the testing protocol is described in detail in [24,25]. The actual protocol is briefly described below. All participants signed an informed consent prior to beginning the study’s procedures. Somatosenory input was altered by modification of the surface: stable (floor) vs. compliant (memory foam or a stability trainer, see below). Visual input was altered by closing the eyes or by several virtual environments as described below. When participants were wearing the Oculus Rift headset, they were standing on a stable force-plate or on two blue soft Theraband® stability trainers (Theraband, OH, USA) placed on top of the force-plate. The two ‘eyes closed’ conditions were done on a stable force-plate and on Airex® memory foam (Sins, Switzerland) placed on top of the force-plate.

The following conditions were applied:

1. Standing on floor with feet together with eyes closed (20 seconds, 2000 data points)
2. Standing on memory foam with feet together with eyes closed (20 seconds, 2000 data points)
3. Display of stars on 3 walls moving medio-laterally at an amplitude of 4 mm and frequency of 0.48 Hz [26] (60 seconds, 6000 data points)
4. Same as (3) but the movement is anterior-posterior at an amplitude of 5 mm, frequency 0.2 Hz [26]
5. Same as (4), amplitude 32 mm [26]
6. A busy street scene with changes in speed of movement and light (120 seconds, 12000 data points) [25]
7. A park scene where the participants were asked to avoid a ball approaching their head (120 seconds, 12000 data points) [25]

Scenes 3 through 7 were performed twice on the floor and twice on the stability trainers when participants are standing hips-width apart. The floor conditions were always introduced first, but the order of conditions within a surface was randomized. In all but the park scene, participants were asked to do whatever felt natural to them to maintain their balance.

### Data recording and processing

Center-of-pressure (COP) fluctuations during the standing postural tasks were recorded by a Kistler (Kistler Group, Winterthur, Switzerland) force-plate at 100 Hz. SampEn was calculated from the COP time-series in the medio-lateral (ML) and anterior-posterior (AP) planes as follows. For the data set (*x*_1_,…,*x*_*N*_) with standard deviation *σ* and parameters (*r*, *m*), let *P* be the number of indices *i* < *j* in {1,…,*N*−*m*} such that *d*(*x*_*i*_…*x*_*i*+*m*_,*x*_*j*_…*x*_*j*+*m*_) ≤ *rσ*, and let *Q* be the number of indices *i* < *j* in {1,…,*N*−*m*} such that *d*(*x*_*i*_…*x*_*i*+*m*_,*x*_*j*_…*x*_*j*+*m*_) ≤ *rσ*, in which *d*(*u*,*v*): = max_*k*_|*u*_*k*_ – *v*_*k*_| denotes the *L*^∞^-distance between vectors. With this notation, SampEn = −log(*P*/*Q*).

### Statistical analysis

For all questions, we employed a sensitivity analysis approach at different *r* values. This sensitivity analysis approach allows us to find the *r* for which differences in SampEn could be detected for the different groups on the different surfaces. To do this, at each value of *r* we fit a linear mixed effects model. Linear mixed effects models estimate the overall difference in SampEn between the floor and the compliant surface data for each group, while accounting for variation on each task [27]. Within each model we tested the main effect of the following factors: Group (patients vs. controls) and Surface: (floor vs. compliant surface). We also fit an interaction between group and surface to assess whether the groups differed in their response to the changing surface. Each model controlled for a fixed effect of Task (each one of the 7 tasks in the protocol) since SampEn values may be inherently different across the different tasks in the protocols, and a random effect for individuals, since there might be inherent differences in individuals’ entropy values and we have repeated measures for each individual.

A main effect of group would indicate that SampEn is significantly different between patients with vestibular dysfunction and control across tasks and surface conditions. A main effect of surface would indicate that SampEn is significantly different between the compliant and stable surfaces across tasks in both groups. A significant group X surface interaction would indicate that the effect of surface (enhancing or decreasing SampEn) differs between the groups. P-values for the main effects of surface and group, representing the average difference in SampEn across surfaces, as well as the interaction term, were calculated through the Satterthwaite approximation for the degrees of freedom for the T-distribution [28].

We ran the same model under the following conditions:

Question 1: how does the model change given different r’s in both ML and AP?

- We kept down-sampling by 2.
- We compared six different *r*’s: 0.05, 0.1, 0.15. 0.2, 0.25, 0.3 (times the standard deviation of the signal) across two different *m*’s (*m* = 2, *m* = 3). Because the patients group was larger and older on average, to eliminate age as a possible confounder, we reran the same model with 12 patients and 11 controls who were completely matched by age, with average age 52.

Question 2: how does the model change given down-sampling or filtering in both ML and AP?

- We kept *r* = 0.1, *m* = 2
- We compared 4 different down-sampling approaches: by 1 (keeping all data points), by 2, 3 and 4; and one filtering approach: a low-pass Butterworth filter with a cutoff frequency at 10 Hz and no down-sampling.

Question 3: how does the model change given differencing and detrending?

- We kept *r* = 0.1, *m* = 2 and down-sampling by 2 (DS = 2)
- We compared the original data series (as analyzed in question 1), to the differenced data series, and the detrended data series

Analysis and plots were done via R 3.4.0 2016, Matlab R2017a, Mathematica 11.

## Results

Overall, we found a consistent large significant main effect of surface (SampEn was lower on the compliant surface) in both the ML and AP planes and no significant main effect of group. Patients and controls had similar levels of SampEn when standing on the compliant surface. Patients had higher ML SampEn when standing on the floor compared with controls, but this difference was not significant. Below is a detailed description of the effect of each signal manipulation on these results.

### Sample entropy given *r* and *m* (DS = 2)

**Fig 1:**
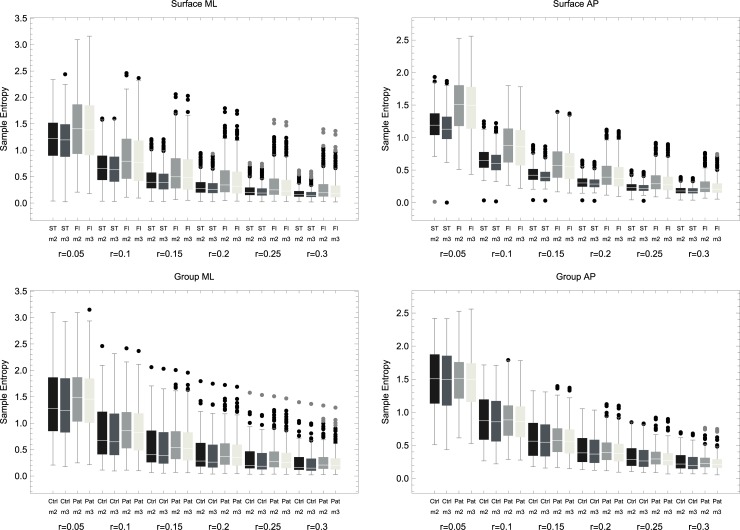
Top: comparison of sample entropy values derived from tasks performed on the stability trainer (ST) or floor (Fl) in the medio-lateral (ML) plane (left) and anterior-posterior (AP) plane (right). This comparison is shown for *m* = 2 and *m* = 3 across 6 values of *r*: 0.05, 0.1, 0.15, 0.2, 0.25, 0.3. Sample entropy was consistently higher on the floor as compared to ST in both planes and for all *m*’s and *r*’s. Bottom: A between-group comparison of sample entropy for tasks performed on the floor. Across *m*’s and *r*’s, patients were consistently higher in the ML plane with no main effect of group but a significant interaction group × surface for *r* = 0.05 and *r* = 0.1. There were no between-group differences on the AP plane.

Fig 1 demonstrates changes in SampEn given surface (top) and group (bottom) across *r*’s and *m*’s. For ML data, a significant main effect of surface remained consistent, whereas an interaction group × surface became less apparent as *r* increased, as follows:

At *r* = 0.05: adjusting for differences in task, we saw a significant main effect of surface (*p* = 0.00143), no main effect of group (*p* = 0.46986), and a significant interaction (*p* = 0.01) indicating that the difference between surfaces is exacerbated for the patients, specifically, patients had a greater decrease from the floor to the compliant surface compared with controls. At *r* = 0.1: adjusting for differences in task, we observed a significant main effect of surface (*p* = 0.0004), no main effect of group (*p* = 0.53), and a significant interaction (*p* = 0.04). As *r* increased, a main effect of surface remained significant (*p* = 0.0002 for *r* = 0.15; *p* < 0.0001 for *r*’s of 0.2, 0.25 and 0.3); there was no main effect of group (*p* = 0.6, 0.67, 0.73, 0.76 for *r*’s 0.15, 0.2, 0.25, 0.3 respectively), and the interaction term was no longer significant with a gradually increasing *p* value (0.1, 0.19, 0.27 and 0.34 for *r*’s 0.15, 0.2, 0.25, 0.3 respectively) although, as can be seen in Fig 1, SampEn of patients remained somewhat higher on the floor across *r*’s. These findings remained consistent when tested with subset of the patient group that was age matched to the control group. Specifically, in the ML direction a significant interaction (group × surface) was noted at *r* = 0.05 (*p* = 0.01) and *r* = 0.1 (*p* = 0.04) and was no longer significant as *r* increased. See S1 Fig in the supplementary material for the analogue of Fig 1 with the age-matched groups.

For AP data, the effect of surface was consistently significant (*p* values around 0) with no main effect of group and no significant interaction regardless of *r*. These findings were the same when running the models with *m* = 3. Namely, the main effect of surface remained with *p* values around 0, and a significant interaction group × surface was noted for *r* = 0.05 (*p* = 0.007) and *r* = 0.1 (*p* = 0.03) in the ML plane.

### Sample entropy given down-sampling and filtering (*r* = 0.1, *m* = 2)

**Fig 2:**
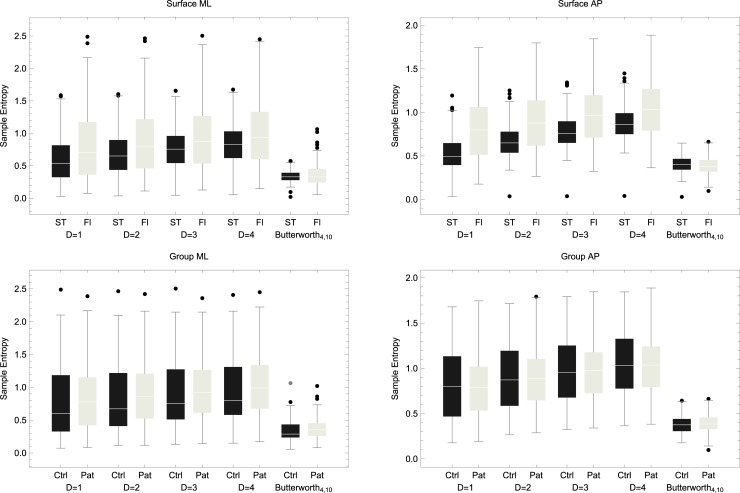
Top: comparison of sample entropy values derived from tasks performed on the stability trainer (ST) or floor (Fl) in the medio-lateral (ML) plane (left) and anterior-posterior (AP) plane (right). This comparison is shown for *m* = 2 and *r* = 0.1 across different levels of down-sampling (DS) by 1, 2, 3 and 4 or when applying a 4^th^ order low-pass Butterworth filter with a cutoff frequency at 10 Hz. Sample entropy was significantly higher on the floor for all DS levels in both planes but not when applying the filter. Bottom: A between-group comparison of sample entropy for tasks performed on the floor given DS or filter in the ML (left side) or AP (right side) plane.

For ML data, a significant main effect of surface was observed for all down-sampling (DS) approaches (DS = 1, *p* < 0.0001; DS = 2, *p* = 0.0005; DS = 3, *p* = 0.0034; DS = 4, *p* = 0.025). A significant interaction group × surface was also observed across down-sampling levels (*p* values between 0.02 to 0.04). As can be seen in Fig 2, there was a mild increase in SampEn values as DS increased, i.e., with less data points. When applying a Butterworth filter and DS = 1, no significant effects were found: no main effect of surface (*p* = 0.91), or group (*p* = 0.252), and no significant interaction (*p* = 0.281). SampEn from filtered data were overall between 0 and 1 whereas SampEn from DS data were between 1 and 3.

For AP data, a significant main effect of surface was maintained across down-sampling levels (*p* < 0.0001) but not when applying the filter (*p* = 0.065). No significant interaction was observed with *p* values around 0.8 for DS data and *p* = 0.34 for filtered data. (See Fig 2).

### The effect of stationarity (DS = 2, *r* = 0.1, *m* = 2)

**Fig 3:**
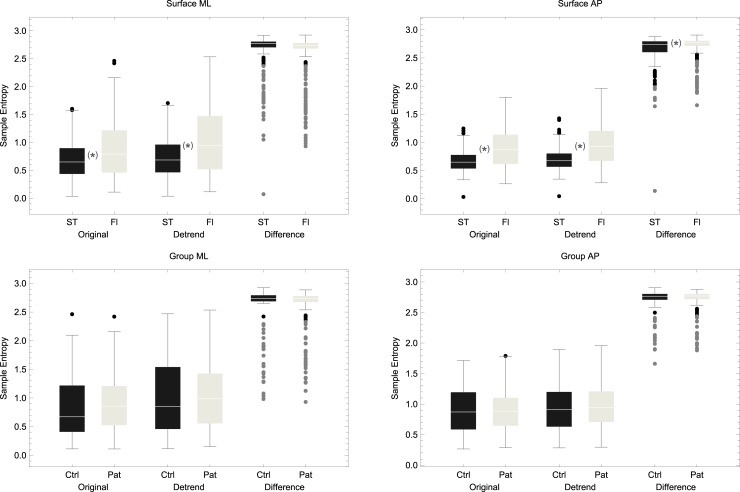
Sample entropy between surfaces (top) and between groups on the floor (bottom) in the ML direction (left hand side) and AP direction (right hand side) for original data, detrended data and differenced data when *m* = 2 and *r* = 0.1.

**Fig 4:**
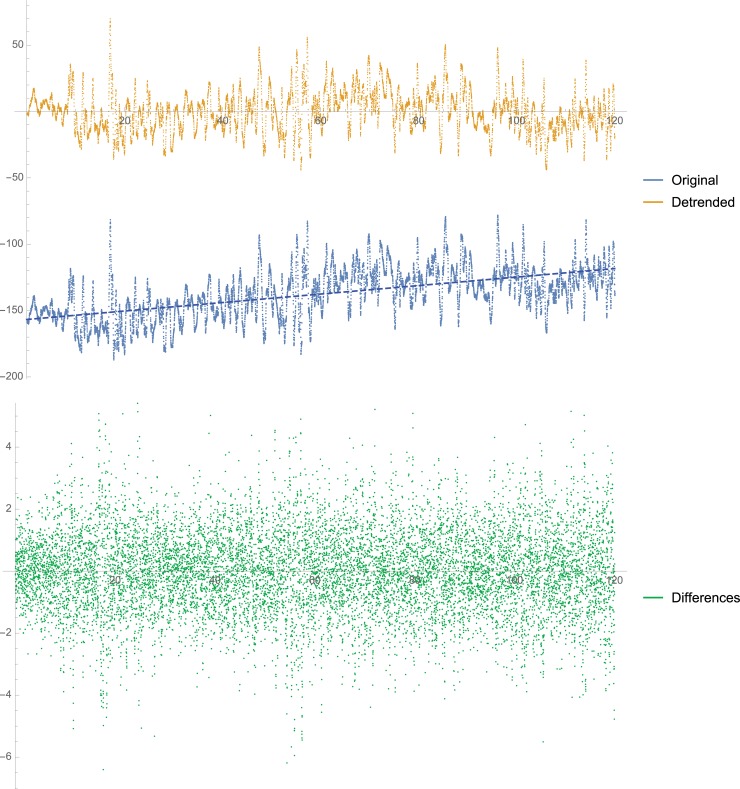
We used the raw anterior-posterior time series (top) from one scene (the park scene, 120 seconds long) performed by a patient when standing on the floor to demonstrate the effect of detrending (middle) and differencing (bottom). The corresponding sample entropy values in this scene are: Raw: 0.463, Detrended: 0.56, Differenced: 2.6.

See Fig 3 for the cumulative effect of detrending and differencing on SampEn. See Fig 4 for a visual demonstration of what each manipulation does to the raw data exemplified in one scene. A main effect of surface in the ML was maintained with detrending (*p* < 0.0001) but not with differencing (*p* = 0.17). In the AP direction, the main effect of surface was significant for all with *p* < 0.0001 for detrending and *p* = 0.04 for the differenced data. Unlike the original data, no significant interaction group × surface was observed in the ML direction for detrended data (*p* = 0.07) or differenced data (*p* = 0.13).

## Discussion

Within this study we applied SampEn as an outcome measure for a virtual reality assessment of sensory integration for postural control in people with vestibular dysfunction. We then investigated the effect of parameter manipulation (*m*, *r*) and signal processing on the following clinical question: is there a difference between groups given the virtual task and the surface? We found that a large significant effect of surface has not changed with increasing *r* whereas a significant group × surface interaction was washed out with values of *r* > 0.1 for both *m* = 2 and *m* = 3. Down-sampling did not affect those differences, but the effect of surface was no longer seen when data were passed through a 4^th^ order low-pass Butterworth filter with cutoff at 10 Hz, and SampEn values were reduced by a factor of 2. In addition, differencing the data eliminated the effects in the ML plane and increased SampEn values by a factor of 5. Detrending weakened the observed differences but SampEn values were similar to those derived from the raw data. In what follows we discuss the methodological and physiological aspects of these results and their implications in postural control research.

**Fig 5:**
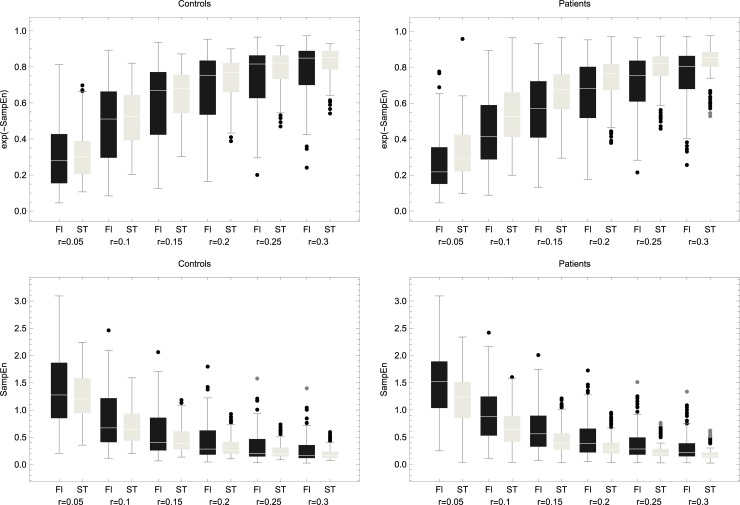
Distributions of SampEn and e^−SampEn^ as a function of the radius of similarity *r* for the data samples of patients and controls on the floor and stability trainer.

In their discussion on the sensitivity of SampEn to the parameters *m*, *r* as well as the data length *N*, Richman and Moorman argued that when the data consist of independent identically distributed (i.i.d.) random variables with sufficiently smooth distributions (e.g., uniform on [0,1], that was highlighted there, noting other distributions, including Gaussian, exponential, and *γ*-distributions, gave essentially identical results), the mean values of SampEn and ApEn are nearly identical, decreasing in proportion to log(*r*) for *r* ≤ 1. They expected that the comparison of SampEn between two data sources should be consistent for different choices of *m*, *r*, at least for large *N*. They further studied the effect of *N* on data consisting of i.i.d. Gaussians, noting a stark difference between numerical estimates of the variance of SampEn with *N* = 15 compared to *N* = 100; they suspected that this phenomenon, dubbed “residual bias,” is due to dependencies between overlapping templates that are non-negligible at small *N*. In our results, consistency across *m*, *r* was found in most of our comparisons, but a significant interaction surface × group in the ML plane became less and less apparent with increasing *r*. One possible explanation is depicted in Fig 5, where the distributions of SampEn for both patients and for controls are seen to become more concentrated upon increasing *r* on the stability trainer compared to their analogs on the floor. Based on theoretical analysis shown below for the i.i.d. case, we suspect that, with a larger data length *N*, this interaction would remain significant also at larger *r*. Indeed, as we next demonstrate, for i.i.d. data the variance of SampEn decreases in proportion to *N*.

For data consisting of i.i.d. random variables *X*_1_,…,*X*_*N*_, write *σ*^2^ = Var(*X*_*i*_). With *P*, *Q* as above (SampEn = −log(*P*/*Q*)), the expected value of *P*/*Q* takes a particularly simple form: if $\tilde{P}$ and $\tilde{Q}$ count only indices *i* < *j* as above excluding *j* = *i* + 1 (the errors $\left|\tilde{P}-P|,|\tilde{Q}-Q\right|$, each at most *N*, are negligible), the mean $E[\tilde{P}/\tilde{Q}]$ is the probability that two independent samples of *X*_*i*_ are within distance *rσ*, which we denote by *g*(*r*). By McDiarmid’s Inequality, the probability that |P−E[P]| exceeds some *a* > 0 is at most 2exp(−*a*^2^/2*N*^3^), and the same holds for $\tilde{P},Q,\tilde{Q}$. That is, *P* concentrates around its mean with exponential tails beyond a deviation of *N*^3/2^ (note that this indeed absorbs the difference between *P* and $\tilde{P}$), as does *Q*, which, since these means are of order *N*^2^ each, translates to a bound of order $1/\sqrt{N}$ on the standard deviation of *P*/*Q*. This implies that –log(*P*/*Q*) is similarly concentrated about *h*(*r*) = −log*g*(*r*) with exponential tails beyond a deviation of $1/\sqrt{N}$, and the variance of SampEn is of order at most 1/*N*.

**Fig 6:**
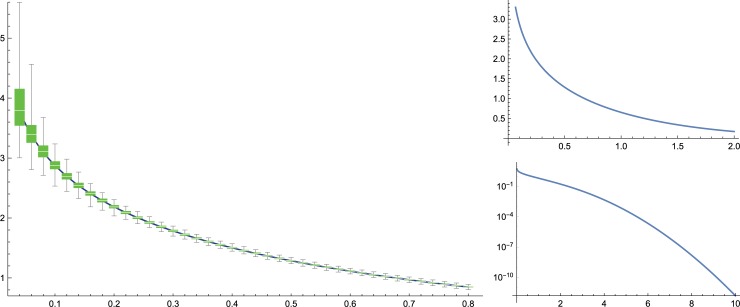
On the left: distribution of SampEn for *m* = 2 and varying *r* on (*X*_1_,…*X*_*N*_) for *N* = 1000 where *X*_*i*_′*s* are i.i.d. standard Gaussians; the mean (blue curve) is $h(r)=-\log\;Erf(\frac{r}{2}).$ On the right: the function *h*(*r*) and its behavior for large *r* (on bottom: in log-scale).

Concretely, consider the case where the *X*_*i*_′*s* are i.i.d. standard Gaussians, for which the residual bias was depicted in Fig 3 of [3]. (Our bound of order 1/*N* on the variance of SampEn provides a rate of decay of that residual bias.) There, *X*_*i*_ − *X*_*j*_ is Gaussian with variance 2, thus *g*(*r*) = Erf(*r*/2), where $Erf(x)=\frac{2}{\sqrt{\pi }}\int_{-x}^{x}e^{-t^{2}}dt$ is the error function. Fig 6 shows the mean and concentration of SampEn for *N* = 1000. Note that, as SampEn is concentrated around −log Erf(*r*/2), it behaves roughly as exp(−(*r*/2)^2^) for large *r*, in contrast to being proportional to log *r* for small *r* as reported in [3].

In our data, down-sampling led to a mild increase in SampEn, i.e., the dataset appears less regular, as was observed in Rhea et al.[19], however, a significant main effect of surface and significant group × surface interactions were maintained across levels of down-sampling (by 2, by 3 and by 4). The observed decrease in SampEn with higher sampling rates is likely due to the fact that, when the sampling rate is well above the frequency of the tested behavior, there is an artificial increase in the number of matches, which leads to a decrease in SampEn [29]. Since the primary frequency of postural sway (in our data and in other studies, see, e.g., [13]) typically lies between 0.15 Hz and 0.4 Hz, with peaks as low as 0.01 Hz and as high as 3 Hz, we believe that a 25 Hz sampling rate was sufficient to detect the time series pattern and better reflects the underlying postural sway pattern. Filtering, however, may remove postural sway components that are part of the postural control process. Indeed, in our case, filtering eliminated all the differences we observed. As suggested by Stergiou [20] and Rhea et al. [19], filtering may artificially create structure in the data series that does not exist and by that largely increase the number of matches in the time series and make the COP signal appear more regular. Note that, SampEn from raw data were about twice as high as those of the filtered data.

Should non-stationarity be addressed when applying SampEn to posture data? In our case, differencing had a strong impact of the data. The actual values of SampEn were about 3 times as high as those produced by the position data. Similarly, Rhea et al. [19] found SampEn to be about 4 times higher with differenced data than with position data. In addition, most of the differences observed with the raw data changed with the differenced data. In addition to enhancing high frequency noise [23], eliminating the position within static posture tasks affects the time series pattern. For example, if a person is leaning forward to their max range, they are less likely to move more forward from this position than they would if they were moving from the center. In such a case, differencing reduces the ability to predict the following data points and leads to a large increase in SampEn (see Fig 4). Detrending, is a milder manipulation to remove drift because it does not ‘forget’ where the signal was and so any change is still relative to where the person was before. Indeed, detrended SampEn values were quite close to those of the raw data. In addition, with detrending, if there is a large drift, it will center it, if the scene is centered already it will not make much of a difference. Nevertheless, non-stationarity is likely inherent in a biological process and a true physiological phenomenon. Therefore, Stergiou suggested that drift or trend should be removed only if there is a strong reason to believe that this drift is not part of the human performance pattern, that is if a calibration error or a device drift have occurred during data collection [20].

This study is limited by multiple statistical tests conducted on a relatively small and diverse sample size. No a-priori power analysis was conducted as this is a first study testing patients with vestibular dysfunction with our newly developed virtual reality protocol. Nevertheless, the primary difference we observed, the reduced SampEn given surface, was highly significant with *p* values around 0 when analyzing the original data and the effect of increased task challenge on SampEn is well documented in the literature [30,31]. We employed a sensitivity analysis where the radius was increased until the additional variability in the measure caused the differences found by the mixed effect model to disappear. We conducted our analysis in a 2-stage approach, first selecting the r value and then testing the down-sampling (DS) technique based on the sensitivity analysis approach. In order to look at the effect of filter, differencing and DS we wanted to explore whether a significant pheromone that we had identified remains or not. Indeed, we saw that the behavior no longer existed under these signal manipulations procedure suggesting that studies that used different signal processing strategies cannot be compared for SampEn values or clinical conclusions. For completeness, we refer the reader to S2 Fig and S3 Fig in the supplementary material where we demonstrate that the changes in SampEn observed with signal processing were consistent across different parameter choices (*m* and *r*). In addition, our protocol was unique with various virtual reality scenes at different lengths (from 20 seconds through 60 seconds to 120 seconds) and 2 different base-of-support positions (feet together for the 20 seconds scene and feet hips-width apart in the longer scenes). Because there may be inherent differences in the different tasks in terms of SampEn values (the mean SampEn may be different) we included task in the model and controlled for it as a covariate analysis. The fact we did not have a control ‘eyes open’ condition but rather an ‘eyes closed’ condition is a limitation as well. While all of our findings could be specific to the data we collected, and the specific protocol used, they call for a closer observation at choice of parameters and signal processing before SampEn can be applied and raise a question regarding comparison between studies that utilized different signal processing approaches.

## Conclusion

When comparing patients with vestibular dysfunction and controls given virtual reality tasks and surface conditions, we found that *m* = 2 and *m* = 3 provided similar results. In addition, as expected, increasing the radius of similarity (*r*) has led to a decrease in average SampEn values. However, since the variance changes as well, modifying the *r* may or may not affect the clinical question, and, in our case, affected the group by surface interaction but not the large main effect of surface. While down-sampling had increased SampEn values (in-line with the literature), it had little effect on the comparisons, and a significant main effect of surface was detected even when down-sampling the data to 25 Hz. Nevertheless, our clinical take-home-message was different when filtering, differencing or detrending was applied. Based on our findings, we suggest that for SampEn to be compared across postural control studies, there needs to be increased consistency, particularly of signal handling prior to the calculation of SampEn. Procedures such as filtering, differencing or detrending could artificially alter the time series pattern and affect SampEn values. If such procedures are performed they should be well justified.

## Supporting information

S1 Fig This Fig is the analogue of Fig 1 for a subset of the patients that is age-matched to the control group. To account for age differences between the groups, here we present the comparison by group and surface with 12 patients and 11 controls who are matched by age, having excluded 5 patients who were 70–80 years old. Note that the findings are the same as in the whole group suggesting that age was not a confounder.
(EPS)

S2 Fig The effect of down-sampling and filtering for all combinations of *m* = 2 or *m* = 3 and *r* = 0.1, *r* = 0.15 or *r* = 0.2. These Figs demonstrate that our findings are supported across a range of commonly used *m’*s and *r*’s.
(EPS)

S3 Fig The effect of differencing and detrending for all combinations of *m* = 2 or *m* = 3 and *r* = 0.1, *r* = 0.15 or *r* = 0.2. These Figs demonstrate that our findings are supported across a range of commonly used *m’*s and *r*’s.
(EPS)

S1 Table Our complete dataset. (CSV)
